# How to Reduce Errors and Improve Transparency by Using More Precise Citations

**DOI:** 10.3389/fcvm.2022.866279

**Published:** 2022-04-29

**Authors:** Ting-Yu Lin, Tsung-Min Hung

**Affiliations:** Department of Physical Education and Sport Sciences, National Taiwan Normal University, Taipei, Taiwan

**Keywords:** transparency, research reliability, research method, reproducibility, preventing fraud

## Definitions of Citation, Reference, and Quotation

Citations and references are essential components of academic articles. Although the terms citation, quotation, and reference are sometimes used interchangeably, clear definitions of these terms are required to facilitate further discussion and to avoid confusion. In this article, we define “citation” as an in-text indication of a book, article, or other source that supports the author's statement; “reference,” which is typically presented at the end of an academic paper, as the detailed information about the source identified in the corresponding in-text citation; and “quotation” as an exact repetition of a section of text in one of the references. Although misspellings of authors' names and the titles of articles in references are common errors, errors in citations are the most problematic because they can be seriously misleading [(1), D2-3]. A high error rate means that readers cannot be sure whether the statements made by the authors are supported by academic research [(2), Pg13]. Therefore, in this article, we propose a slight modification to the commonly used citation format to improve transparency and reduce the spread of incorrect information.

## The Prevalence of Citation Errors

Jergas and Baethge [(2), T1-2] conducted a systematic review of studies that analyzed citation errors and concluded that in the field of medicine, about 22% of citations were problematic and about 12% were seriously incorrect. A subsequent methodological review by Mogull [(3), R] reported that about 14.5% of citations in medical studies were erroneous, and about two-thirds of these improper citations were considered major errors that contradicted, were unrelated to or failed to substantiate the assertions made. Moreover, the estimated rate of citation errors by Mogull was nearly identical to the 15% error rate reported by de Lacey et al. [(1), D3] more than two decades ago, suggesting that merely pointing out how prevalent these errors do little to reduce them. Therefore, action to remedy this problem is essential.

## A Proposal for Improving Transparency and Reducing Errors

Table 1 provides examples of how to incorporate the location of specific information within a cited paper into some common citation styles. Evaluating and revising existing citation or reference styles is far beyond the scope of this article; therefore, we merely modify these styles by adding information. Writers adopting this method can add just a few letters after each citation, probably via citation management software, without the need to change their familiar citation style. An example can be found in an article published recently by our group (4).

**Table 1:** Comparison of traditional and two recommended citation methods.

**Methods**	**Only articles (traditional)**	**Page(s) and display item(s)**	**Section(s), paragraph(s), and display item(s)**
Examples			
Superscript	^1^	1^(Pg10, F5)^	1^(R5, F5)^
Numbered	(1)	[(1), Pg5–6]	[(1), R3–4]
Author-Date	(Alex et al. 2021)	(Alex et al. 2021, Pg2-3, T1)	(Alex et al. 2021, I3-5, T1)
Recommended or not recommended (see main text)	Not recommended	Recommended	Recommended

*Pg, page; Display item: F, figure; T, table; B, box; Section and paragraph: A, abstract; I, introduction; M, methods; R, results; D, discussion; Arabic numbers after the abbreviation of the section: paragraphs in the given section; S, section; par (used when necessary): paragraph(s), e.g., (Alex et al., 2021, S3.3par2); Other: Sup, supplementary*.

In Table 1, the indicators (i.e., page or section) are used to locate information and to help readers find the relevant information. Hence, the most useful information to include after the traditional in-text citations depends on the context. Flexibility is desirable, because the most precise way may not be the most effective if it confuses readers. For instance, when counting paragraphs is likely to be tiresome (e.g., in a multi-page section), simply citing the page number may be more efficient. However, when a scientific paper has multiple versions with different typesetting (e.g., one from National Center for Biotechnology Information and one from Elsevier), citing the section heading with paragraph numbers (e.g., R3–4 indicates the third and the fourth paragraphs of the Results section) or subsection number (e.g., S3.3) can prevent confusion.

The list of abbreviations in Table 1 for the sections: introduction, methods, results, and discussion, are provided for biomedical-related domains. Therefore, for writers from other disciplines, the abbreviations can be modified as needed. It should be noted that some citation systems in which the page numbers are in demand already exist. For example, the journal *Leonardo* [(5), S“References and Notes”] requires authors to cite page numbers in the reference and a single article can present as different numbers in the in-text citation, e.g., (1) refers to article A pages 2–4 and (2) refers to pages 5–10 in the same article. Although each one encourages precise citation of information location, the above-mentioned systems require the modification of references while our proposal recommends providing the information in in-text citations.

## Discussion

Our proposal is partly motivated by an enlightened article by Bareket et al. (6); however, there are some notable differences. The most prominent difference is that Bareket et al. [(6), Pg2] recommended modifying the reference by adding information such as the study design and sample size, whereas we only suggest slightly modifying the in-text citations. They also suggest specifying whether the reference is a primary study [(6), Pg2–3]. There is no doubt that their method can provide useful information for readers, but incorporating this much information in references can be burdensome and difficult to achieve with commonly used citation managers such as Endnote. Therefore, we suggest only adding location information after the in-text citations because we believe that to make significant progress, it is necessary to find a balance between the burden on writers of incorporating additional information and the ease with which readers can verify the information. The limitation of our proposal is that this method has not yet been tested by empirical study. Further studies are required to test whether adopting our proposal reduces the citation error rate as we expect it might.

## Conclusion

History has shown us that noting the prevalence of citation errors without taking action will not resolve the problem. Here, we propose a more precise citation method that balances the benefit for scientific integrity against the cost for writers, in the hope that this will improve the transparency and accuracy of future research.

## Author Contributions

T-YL wrote and was responsible for the manuscript content. T-MH was responsible for reviewing the manuscript and making final decisions about this article. Both authors contributed to the article and approved the submitted version.

## Funding

T-YL is supported by the Scholarship Program for Elite Doctoral Students [Ministry of Science and Technology (Taiwan)]. The supporter had no role in this protocol and will not influence the implementation or publication of this article.

## Conflict of Interest

The authors declare that the research was conducted in the absence of any commercial or financial relationships that could be construed as a potential conflict of interest.

## Publisher's Note

All claims expressed in this article are solely those of the authors and do not necessarily represent those of their affiliated organizations, or those of the publisher, the editors and the reviewers. Any product that may be evaluated in this article, or claim that may be made by its manufacturer, is not guaranteed or endorsed by the publisher.
